# Initial Outcomes of Zap-X Stereotactic Radiosurgery for the Treatment of Spinal Tumors: A Case Series

**DOI:** 10.7759/cureus.87692

**Published:** 2025-07-10

**Authors:** Patrick Pema, Paxton Sweeney, Harshal Shah, Michael Chaga, Wenzheng Feng, Darra Conti, Jing Feng, Tingyu Wang, Ma Rhudelyn Rodrigo, Joseph Hanley, Timothy Chen, Shabbar Danish

**Affiliations:** 1 Neurosurgery, Hackensack Meridian Health, Hackensack, USA; 2 Neurosurgery, Hackensack Meridian School of Medicine, Hackensack, USA; 3 Neurosurgery, Jersey Shore University Medical Center, Neptune, USA; 4 Radiation Oncology, Jersey Shore University Medical Center, Neptune, USA; 5 Neurological Surgery, Jersey Shore University Medical Center, Neptune, USA

**Keywords:** meningioma, schwannoma, spinal metastasis, stereotactic radiosurgery, zap-x® radiosurgery

## Abstract

Stereotactic radiosurgery (SRS) is an established modality for managing spinal tumors, offering targeted radiation while preserving adjacent critical structures. The ZAP-X gyroscopic SRS system (ZAP Surgical Systems, Inc., San Carlos, CA) introduces a novel, self-shielded, non-isocentric platform that may expand access to SRS and streamline treatment delivery. This study presents initial clinical outcomes and technical feasibility of ZAP-X in treating spinal tumors.

Three patients with spinal tumors, including schwannoma, meningioma, and spinal metastasis, were treated using ZAP-X SRS between February and June 2024. All patients underwent single-session treatment with no acute complications or radiation-related side effects. One patient with spinal metastasis and advanced comorbidities experienced significant pain relief, and another demonstrated a complete resolution of neurological symptoms with no radiographic tumor progression at three- and five-month follow-up. The third patient expired due to unrelated causes, but tolerated treatment without complications.

Early experience suggests that ZAP-X SRS is a safe, accurate, and effective option for treating spinal tumors. Its compact design and streamlined workflow offer potential advantages in both definitive and palliative care settings. Further investigation in larger cohorts with extended follow-up is warranted to validate long-term efficacy and broader clinical utility.

## Introduction

Stereotactic radiosurgery (SRS) is a non-invasive treatment that delivers focused radiation to spinal tumors while minimizing exposure to the surrounding healthy tissue. Its use is particularly relevant in the spine, where nearby critical structures limit surgical options. SRS has been shown to achieve long-term tumor control for benign spinal tumors such as schwannomas and meningiomas, with reported local control rates exceeding 90% at 10 years [1]. It is also used in the management of metastatic spinal disease, where it has been associated with pain relief, preservation of neurological function, and improved tumor control, particularly in cases where surgery is not an option or when tumors are radioresistant [2,3]. SRS is also an option for treating primary spinal sarcomas, especially when achieving clear surgical margins is difficult [4]. While SRS offers a targeted approach, conventional systems such as CyberKnife and Gamma Knife require complex planning and specialized radiation vaults, which may limit accessibility and require large installation and implementation costs [5-7]. The ZAP-X SRS system (ZAP Surgical Systems, Inc., San Carlos, CA) aims to improve on these shortcomings while delivering efficient and effective treatment.

The ZAP-X system was designed to address limitations of current SRS systems. Its self-shielding, gyroscopic design removes the need for a dedicated radiation vault, allowing for treatment in a broader range of clinical settings [8,9]. The system has been studied for its dosimetric accuracy, particularly in small-field treatments, and has been reported to provide consistent radiation delivery to irregularly shaped spinal tumors [10]. Early clinical data indicate that ZAP-X has been used in the treatment of both metastatic and benign spinal tumors, including meningiomas and schwannomas, with reported outcomes comparable to conventional SRS platforms [9]. Furthermore, current literature supports favorable dosimetry of the ZAP-X system, with several early studies achieving comparable or even superior dose conformity, gradients, monitor units, treatment time, and organ-at-risk sparing when compared to existing SRS platforms [11-13]. These studies also encourage further evaluation to determine the role of ZAP-X in routine clinical practice, particularly in comparison to existing SRS systems.

This study examines the clinical use of ZAP-X for spinal tumors, focusing on treatment accuracy, safety, and workflow considerations. By analyzing patient outcomes and technical parameters, these findings may help clarify how ZAP-X fits within the current landscape of spinal SRS and whether its features offer meaningful advantages in specific clinical scenarios.

## Case presentation

Three patients with spinal tumors were treated using the ZAP-X gyroscopic SRS system at Jersey Shore University Medical Center (JSUMC) between February and June 2024. Written informed consent was obtained from all three participants. The study protocol was approved by the Hackensack Meridian Health Institutional Review Board (Pro2024-0029). Data extraction included patient demographics and symptoms, tumor characteristics and diagnosis, radiation dosing and parameters, and postoperative outcomes.

Patient-specific quality assurance

Implementation of patient-specific quality assurance (PSQA) is an essential part of SRS treatment efficacy and accuracy. Before treatment and for each case, a PSQA program was utilized to ensure high accuracy of the planned versus delivered dose. To ensure delivery of the prescribed dose, validation of the dose per monitor unit (MU) is necessary. To verify these calculations, in-house developed software conducts a secondary check to calculate MUs. ZAP-X beam data was calculated, including cone factors, tissue phantom ratios, and off-center ratios with a tolerance of ≥95% maximum dose accuracy. Absolute planar dose measurements were performed using SRS MapCHECK (Sun Nuclear Corporation, Melbourne, FL) where plans were simulated in the ZAP-X treatment planning system (TPS) using the following options: “Simulate plan to ion chamber target center”; 1.2 g·cm^3^ density override for dose calculation; dose grid 0.5 mm; centering the target on the diode detector. Gamma analysis was performed using the SNC Patient software (Sun Nuclear Corporation, Melbourne, FL) with 6FFF angular correction, low-dose threshold of 10%, distance-to-agreement of 1%, dose difference of 2%, and gamma passing rate criteria ≥95%. All three patient treatment plans achieved a secondary check maximum dose accuracy of 99.8%. Gamma passing rate ranged from 95% to 97.4% for all three patient treatment plans.

Machine quality assurance

Quality assurance (QA) of the ZAP-X platform is ensured through the performance of daily QA tests by the Medical Physics team at JSUMC. A description of this procedure is as follows. First, to ensure stability, the system warms up to a level of 6000 monitor units. Subsequently, a Winston-Lutz test is performed. This test utilizes a 4 mm tungsten ball placed into a Hayes Phantom, which is inserted into a patient immobilization mask and placed onto the headrest. Beams are then delivered isocentrically using a 10 mm collimator at six gantry angles. MV images are captured during the beam delivery to measure the offset between the ball’s shadow and the radiation field’s center. Through six exposures, and using specific equations for system geometry, the ball’s position and the beam’s central axis relative to the mechanical isocenter are calculated. Notably, offsets must be less than 1 mm. Second, verification of radiation output at the isocenter must take place. To achieve verification, a PTW TN31021 ion chamber is placed at the machine’s isocenter with the gantry in the 0 degree position. Through a 25 mm collimator, a radiation output of 500 MU is delivered twice; the average reading is used to calculate the output dose. Through this procedure, the measured dose was verified to be within 2% of the actual dose and the dose difference between the primary and secondary monitor chambers was <3%. These tests and calculations ensure accurate radiation dosage and confirm system reliability.

Pre-treatment imaging and treatment planning

**Figure 1:**
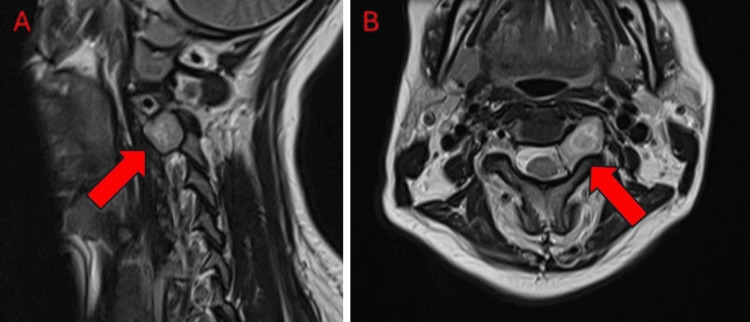
ZAP-X treatment planning MRI images of Patient 1 T2-weighted MRI images of Patient 1, sagittal (A) and axial (B) sections, showing 2.1x1.2x1.6 cm schwannoma. Red arrows indicate cervical schwannoma.

**Figure 2:**
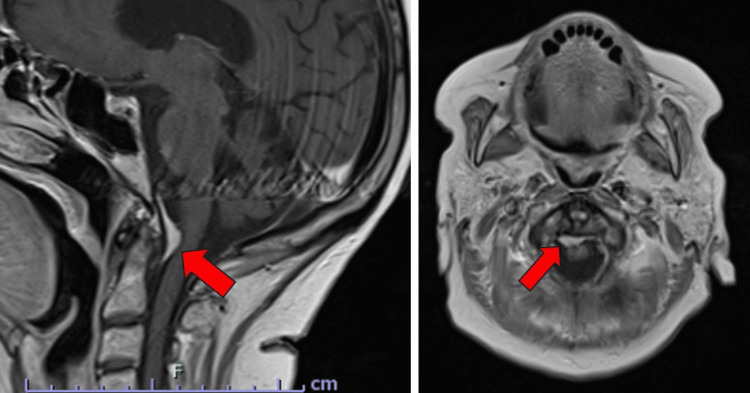
ZAP-X treatment planning MRI images of Patient 2. T1-weighted, contrast-enhanced MRI images of Patient 2, sagittal (A) and axial (B) sections, showing 1.3×0.7×2.6 cm meningioma impinging on the cervical spinal cord. Red arrows indicate cervical meningioma.

**Figure 3:**
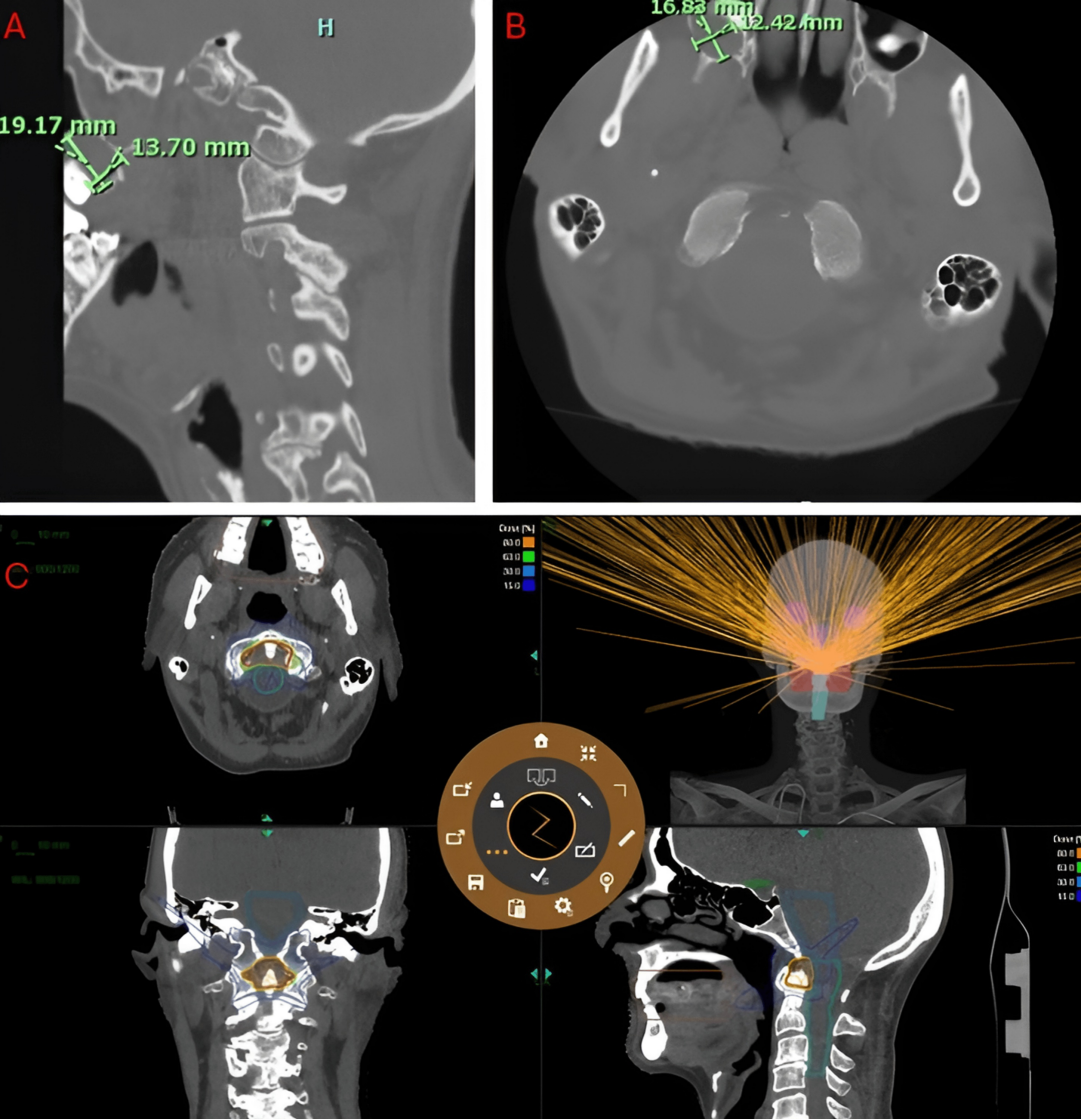
ZAP-X treatment planning images of Patient 3. A,B: CT scan (bone window) showing metastasis to the C2 vertebral body with marrow replacement; C: ZAP-X TPS contours and dose delivery visualizations.

All three patients underwent preoperative imaging. Patients 1 (Figure 1) and 2 (Figure 2) also underwent magnetic resonance imaging (MRI) using a 0.5 T Synaptive MRI (Synaptive Medical, Toronto, Canada). Patient 3 could not receive an MRI because of having an implantable device incompatible with the MRI field. Thus, Patient 3’s treatment planning was done utilizing CT scans (Figure 3). Imaging was then delivered to a treatment planning team consisting of a radiation oncologist, neurosurgeon, and medical physicists who utilized those scans to perform contouring of tumor volumes. The contoured images were confirmed by all treatment planning team members and uploaded to the ZAP-X TPS, version 1.8.58 and 1.8.59. Doses were limited to the spinal cord, brainstem, eyes, optic nerves, optic chiasm, and cochleae based on Timmermann organ-at-risk recommendations [14]. Plan quality was evaluated for conformity and dose fall-off using conformity index (CI) and gradient index (GI).

Case 1

Patient 1 was a 40-year-old woman who presented to the clinic with left extremity numbness, tingling, and weakness as well as a paresthesia in the left posterior auricular region. Initial imaging revealed an enhanced soft tissue mass within the left side of the C2-C3 spinal foramen measuring 1.8x1.2x1.6 cm. An MRI conducted in February 2024 showed that the tumor volume had grown to 2.1x1.2x1.6 cm; Radiology identified this tumor as a C2/C3 schwannoma. Although the tumor was in close proximity to the spinal cord, there was no significant mass effect.

**Figure 4:**
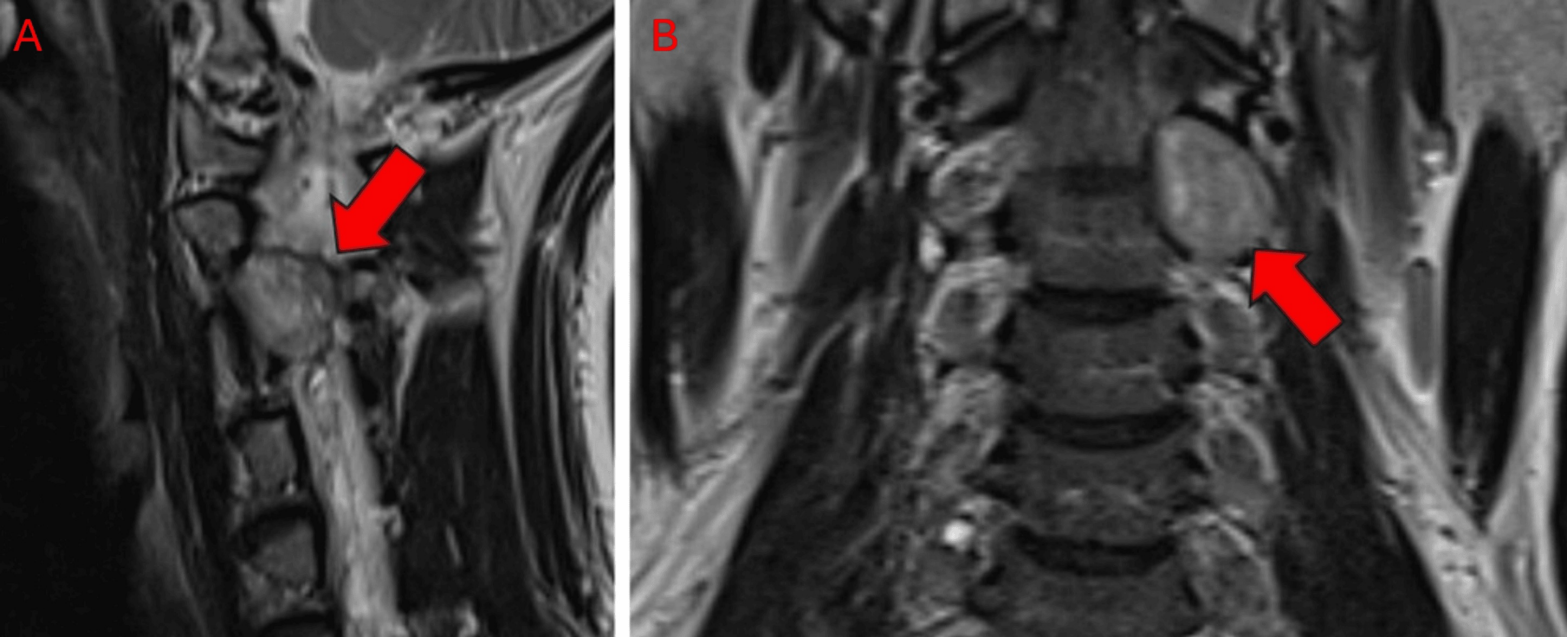
Postoperative MRI imaging of Patient 1. T2-weighted image, sagittal (A) and coronal (B) sections displaying C2 schwannoma with no progression post-SRS treatment with ZAP-X. SRS: Stereotactic radiosurgery.

The patient had a target volume of 3 cm^3^. The patient was placed onto the DP-1009 headrest in mold care and a thermoplastic Efficast head and shoulder mask (Orfit Industries, Jericho, NY). She underwent treatment consisting of 24 Gy in three fractions at a prescription isodose line of 56%, with eight isocenters and 212 beams; the average treatment time was 40 minutes per fraction. Treatment achieved a 98% coverage, a CI of 1.158, and a GI of 2.987. She tolerated her ZAP-X treatment well and experienced no complications or acute adverse events as a result of treatment. At three months postoperatively, she reported complete resolution of paresthesia of her left face and arm with minimal weakness. She reported no neck pain and was able to resume her daily activities. A follow-up MRI (Figure 4) showed no tumor progression.

Case 2

Patient 2 was an 83-year-old woman with a medical history of diffuse large B-cell lymphoma on chemotherapy, type 2 diabetes mellitus, and hypertension who presented with intermittent slurred speech, fatigue, and an unsteady gait. Initial evaluation, including a non-contrast head CT and MRI, identified a 1.3×0.7×2.6 cm enhancing lesion at the craniocervical junction, consistent with a meningioma, causing mild effacement of the upper cervical spinal cord. Neurological symptoms included progressive dysarthria, balance dysfunction, and recurrent falls, resulting in a right lower extremity bimalleolar fracture. Despite no immediate evidence of acute ischemia or vasogenic edema, her clinical course suggested that the tumor contributed to her gait instability.

The patient had a target volume of 0.61 cm^3^. The patient was placed onto the DP-1009 headrest in mold care and a thermoplastic Efficast head and shoulder mask. She underwent treatment consisting of 30 Gy in five fractions at a prescription isodose line of 66%, with 16 isocenters and 280 beams; the average treatment time was 54 minutes per fraction. Treatment achieved a 97.35% coverage, a CI of 1.167, and a GI of 3.536. The procedure was well-tolerated, with no immediate complications reported, and the patient was discharged in a stable condition. Unfortunately, at one month post-treatment, the patient experienced an episode of severe sepsis complicated by diabetic ketoacidosis and expired.

Case 3

Patient 3 was a 59-year-old man with a history of coronary artery disease, heart failure, atrial flutter, pneumonia, thyroid disease, hypoalbuminemia, prostate cancer, and colon polyps who presented with progressive neck pain. He was treated for thyroid cancer in the early 1990s with surgery, chemotherapy, and radiation. His oncologic history also includes chemotherapy (ABVD x6), chest radiation, and multiple surgical interventions. He presented to radiation oncology after CT revealed prostate cancer metastases extending from the top of C1 up towards the medulla and coming within 5 mm of the brainstem. CT imaging showed sclerotic changes and extensive marrow replacement in the C2 vertebral body. The vertebral height was preserved, and there was no evidence of spinal nerve involvement. Given his comorbidities and prior treatments, open surgery was not considered a viable option. 

Patient 3 had an automatic implantable cardioverter-defibrillator (AICD), which is not compatible with MRI, necessitating the use of CT images for treatment planning and tumor contouring. The patient had a target volume of 6.29 cm^3^. The patient was placed onto the DP-1009 headrest in mold care and a thermoplastic Efficast head and shoulder mask. He underwent treatment consisting of 30 Gy in five fractions at a prescription isodose line of 63%, with 13 isocenters and 357 beams; the average treatment time was 57 minutes per fraction. Treatment achieved a 96.19% coverage, a CI of 1.066, and a GI of 2.578. Patient 3 tolerated the procedure well with no side effects or complications related to SRS. Upon follow-up at five months post-operatively, he reported complete resolution of neck pain and was able to resume his daily routine without limitations.

## Discussion

Our report of these three cases aims to inform the current literature surrounding SRS treatment for spinal tumors and to highlight the efficacy of the ZAP-X system for this specific pathology. In today’s current medical environment, SRS is a mainstay of treatment for both benign and malignant spinal lesions [2,11,12]. This treatment with SRS has proven to be effective while minimizing exposure to the spinal cord [11,13]. Here, we aim to highlight the safety and efficacy of the ZAP-X system by demonstrating minimal toxicity during treatment, efficient and accurate dose delivery, and its excellent side effect profile. 

A common challenge of SRS treatment is radiation toxicity to adjacent organs. Literature currently indicates that even small deviations in dosage delivery can result in damage to organs surrounding the targeted tissue [15]. As such, accuracy of dosage delivery is of critical importance. In our cases, high treatment delivery accuracy was ensured through both high-precision machine calibration, onboard imaging, and utilization of a Nanor head and Efficast head and shoulder immobilization masks (Orfit Industries, Jericho, NY). At follow-up, no radiation toxicity was reported or observed. SRS therapy can also result in radiation-related complications in the following months after treatment. Adverse radiation effects (AREs) are symptoms that may occur in the months following an SRS procedure; patients may experience fatigue, vertigo, headache, or gait instability. Our three patients experienced neither immediate side effects or AREs. While further follow-up is needed for these patients, these preliminary results show a promising side effect profile.

We also demonstrated that ZAP-X treatment is feasible even in cases where MRI imaging cannot be obtained due to the presence of an implantable device. In such cases, CT imaging may be used to guide dose delivery. The CT images must be taken with the patient in the exact immobilization position and thin slice images are necessary to ensure accuracy [16]. Treatment for Patient 3 involved such steps and resulted in excellent dose accuracy while maintaining homogeneity and gradient indices. This result proves that CT images may be used safely and effectively in ZAP-X treatment planning. 

Treatment with SRS can be an important staple of pain management in terminal patients with bony metastasis [17,18]. While Patient 2 did not have the chance to complete any follow-up visits, her treatment parameters and details can still inform the use of SRS for meningiomas of the cervical spine, specifically regarding its use in an end-of-life pain relief context. Current literature supports the use of SRS platforms in this manner and SRS has been found to be superior to conventional external beam radiotherapy for palliative purposes [19,20]. A study by Ito et al. demonstrated that SRS therapy provided rapid and significant pain relief from spinal metastases, as well as decreasing patients’ opioid requirements while preserving cognitive function [19]. Similarly, Wang et al. reported a significant reduction in both pain and opioid use in patients with spinal metastases [12]. Our results with Patient 2 can contribute to the fine-tuning treatment parameters for use in pain relief and palliation. She tolerated her treatment well with no side effects or complications, demonstrating the feasibility of ZAP-X therapy in a palliative setting. 

SRS therapy is also important to ensure local control of spinal tumors. Current SRS modalities show excellent tumor control rates, often exceeding 90%, while minimizing AREs and long-term complications [12,21,22]. ZAP-X achieved similar preliminary results; patients 1 and 3 showed no tumor progression on MRI at most recent follow-up. Thus, the ZAP-X system demonstrated excellent local tumor control and further follow-up is needed to gauge its effectiveness to that end.

Limitations

The ZAP-X gyroscopic SRS system is a relatively new platform, with only six sites currently operating in the United States [23]. The first ZAP-X Machine was installed in 2019; thus, long-term clinical data is lacking surrounding this machine [24]. Further studies should aim to gauge treatment effectiveness as measured by progression-free survival, tumor control, pain reduction, and more over longer intervals, such as five or 10 years. Our study can provide only short-term clinical effectiveness and feasibility at this time.

In future studies, care must be taken to account for the mobile nature of the cervical spine when assessing dose conformity and accuracy. Since immobilization takes place primarily through the skull, any target outside of that region may possess higher mobility during treatment [25]. To this end, ZAP-X treatment can utilize the Efficast immobilization mask, which extends to the neck region and is rated for immobilization of C1-C3 vertebrae. However, the efficacy of this mask for complete immobilization of the upper cervical spine has yet to be assessed through large-volume studies. Additionally, a large-study analysis of the Efficast mask and its efficacy for cervical spine immobilization beyond those vertebrae is lacking. Further dosimetric data is needed to elucidate the precise accuracy of the ZAP-X system and Efficast mask when targeting the cervical spine. With these challenges in mind, at this time, we only endorse the feasibility ZAP-X treatment for lesions of the upper C1-C3 cervical spine.

## Conclusions

SRS is an established treatment modality for spinal tumors, with the ability to offer targeted radiation while sparing adjacent critical structures. In this case series, all patients tolerated ZAP-X SRS spinal tumor treatment without experiencing acute or delayed radiation-related side effects, underscoring the safety of the system. Although one patient expired due to unrelated medical complications, their case highlights the potential use of ZAP-X in palliative care patients with spinal metastases. Preliminary local control results were excellent. Further studies with larger cohorts and extended follow-up are necessary to better define the role of ZAP-X in the treatment of spinal lesions.
